# Lactate clearance as a determinant of mortality in surgical patients

**DOI:** 10.1186/cc12154

**Published:** 2013-03-19

**Authors:** FF Amorim, EB Moura, AR Santana, FB Soares, LG Godoy, TA Rodrigues, LJ Almeida, GM FIlho, TA Silva, AP Amorim, JA Neto, MO Maia

**Affiliations:** 1 Escola Superior de Ciências da Saúde, Brasília, Brazil; 2 Unidade de Terapia Intensiva Adulto do Hospital Santa Luzia, Brasília, Brazil; 3 Liga Academica de Medicina Intensiva do Distrito Federal (LIGAMI), Brasilia, Brazil

## Introduction

Serial measurements of lactate over time may be a better prognosticator than a single lactate concentration [1]. Early lactate-guided therapy also reduces ICU length of stay and ICU and hospital mortality [2]. This study aims to assess the prognostic value of the lactate clearance (LC) in the first 24 hours in surgical patients.

## Methods

In a prospective cohort during 1 year, we followed consecutively enrolled patients admitted immediately postoperative to the surgical ICU of Hospital Santa Luzia, Brasília, Brazil. Patients were assigned to two groups: LC >10% and LC ≤10%. The primary outcome measure was mortality at 7 and 28 days. The secondary outcome included hospital and ICU length of stay (LOS).

## Results

A total of 417 patients were followed. In total, 50.4% were male and 83% underwent elective surgery. The mean age was 59 ± 16, APACHE II score 8 ± 5, SAPS 2 26 ± 11. The mortality at 7 days was 0.95% (*n = *4) and the mortality at 28 days was 2.15% (*n = *9), respectively. Hospital mortality was 4.79% (*n = *20). Sixty-one percent (*n = *255) of the patients had LC >10% versus 39% (*n = *162) with LC ≤10%. Those who had LC ≤10% were older (62 ± 16 vs. 57 ± 17, *P *= 0.00) and had greater APACHE II score (9 ± 6 vs. 7 ± 4, *P *= 0.00) and SAPS 2 (28 ± 12 vs. 25 ± 10, *P *= 0.02). There was no difference in ICU LOS (5 ± 12 vs. 4 ± 9 days, *P *= 0.54) and hospital LOS (10 ± 15 vs. 9 ± 11 days, *P *= 0.48). Initial lactate levels were lower in the group with LC ≤10% (1.1 ± 0.9 vs. 1.9 ± 1.6, *P *= 0.00); however, mean lactate was higher in 24 hours (2.0 ± 1.8 vs. 1.0 ± 0.7, *P *= 0.00). All of the patients who died in the first 7 days had LC ≤10% (2.46%, *n = *4, *P *= 0.02); this group also had a higher mortality at 28 days (4.32%, *n = *7 vs. 0.78%, *n = *2; *P *= 0.03). The relative risk for mortality LC ≤10% in 7 and 28 days was 1.02 (95% CI: 1.00 to 1.05) and 5.07 (95% CI: 1.17 to 27.09), respectively. Significant difference was observed in the Kaplan-Meier survival curves for 7 and 28 days (*P *= 0.01 and 0.02, respectively). The sensibility of LC ≤10% was 100% (95% CI: 51 to 100%) for 7-day mortality and 78% (95% CI: 45 to 94%) for 28-day mortality. The specificity was 62% (95% CI: 57 to 66%) for 7-day mortality and 62% (95% CI: 57 to 66%) for 28-day mortality.

## Conclusion

Despite initial lactate levels, lactate clearance ≤10% proved to be a good predictor of mortality in 7 and 28 days in surgical patients admitted in the postoperative period to the ICU.
